# Supplementary material for: Willow Aboveground and Belowground Traits Can Predict Phytoremediation Services
Source: Plants (Basel). 2021 Sep 2;10(9):1824. doi: 10.3390/plants10091824 (PMC8471398; doi:10.3390/plants10091824)
Supplement: Supplementary file 1 [file plants-10-01824-s001.zip › plants-1325108_MAT_SUP_clean.pdf]

Willow aboveground and belowground traits can predict phytoremediation services

Supplementary figures

Béatrice Gervais-Bergeron[1*], Pierre-Luc Chagnon[1] et Michel Labrecque[1]

1. Institut de recherche en biologie végétale, Université de Montréal, 4101 Sherbrooke East, Montréal, Québec, H1X 2B2, Canada

Table S1. Trait values measured in this study from all plantations and from the Global TRY database (mean species values) for the three willow species used in this study.

Specie	SLA		LDMC		LNC	
	Measured range	TRY	Measured range	TRY	Measured range	TRY
Salix purpurea		11.9		396.8		2.6
Salix miyabeana	12.4 – 18.5	NA	288.8 – 352.8	NA	2.2 – 3.0	2.4
Salix gmelinii		17.5		277.1		2.5

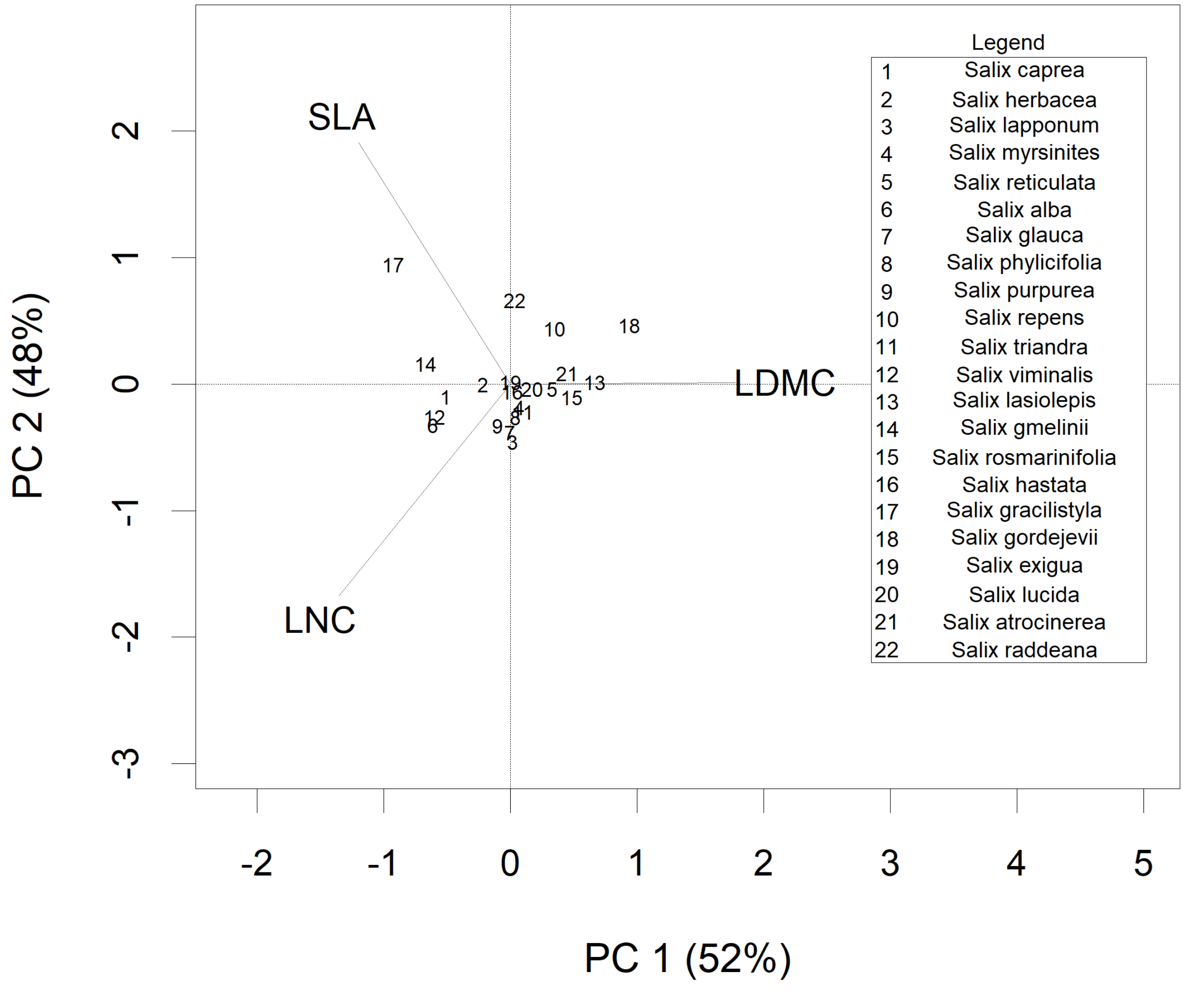

Figure S1. Principal component analysis (PCA, distant biplot) illustrating the functional distinction between 22 *Salix* spp. according to their available values of specific leaf area (SLA), leaf nitrogen content (LNC) and leaf dry matter content (LDMC) on the global TRY database.

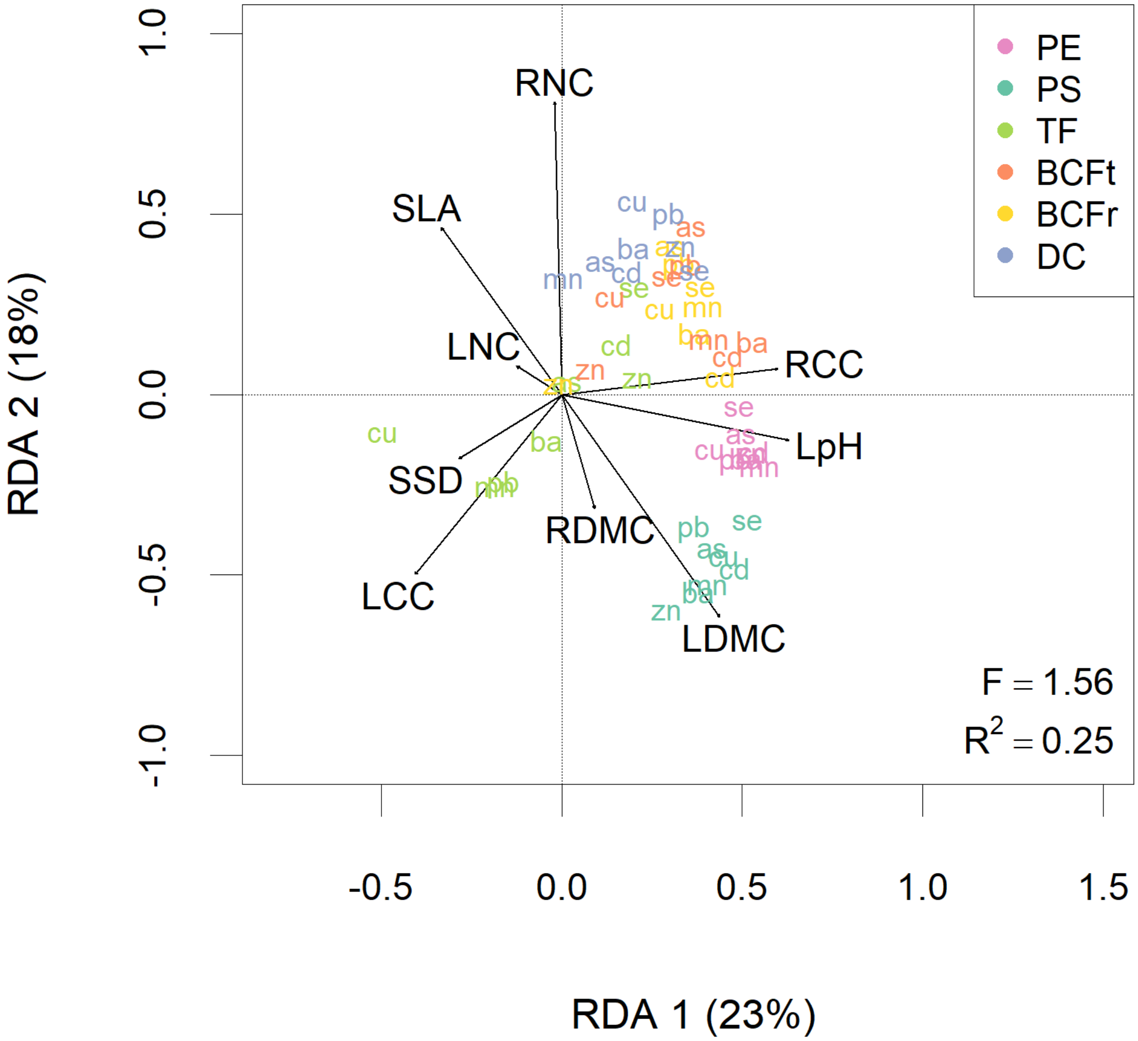

Figure S2. Redundancy analysis (RDA) regressing the six phytoremediation services for every TE (As, Ba, Cd, Cu, Mn, Pb, Se, and Zn) to the functional traits. The colour of the TE is associated with each service from the legend. *Abbreviations:* Phytoextraction (PE), Phytostabilization (PS), Translocation factor (TF), Total bioconcentration factor (BCFt), Root bioconcentration factor (BCFr), Soil decontamination (DC), leaf area (LA), specific leaf area (SLA), leaf pH (LpH), leaf and root dry matter content (LDMC and RDMC), leaf nitrogen and carbon content (LNC and LCC), root nitrogen and carbon content (RNC and RCC) and specific stem density (SSD).

Table S2. Mean services rendered by willow plantations for every TE measured on the brownfield at the end of the fourth growing season (2019) and their standard deviation (SD).

Service units	PE (g/ha)	SD	PS (g/ha)	SD	TF	SD	BCFr	SD	BCFt	SD	DC (%)	SD
As	2.06	1.86	0.03	0.01	0.23	0.12	0.11	0.04	0.13	0.04	0.05	0.44
Ba	348.98	293.16	1.56	0.68	0.63	0.17	0.18	0.04	0.28	0.04	0.17	0.16
Cd	27.62	23.03	0.03	0.01	2.60	0.49	0.49	0.1	1.74	0.41	-0.09	0.44
Cu	238.99	144.91	2.96	1.23	0.31	0.22	0.51	0.18	0.65	0.2	-0.31	1.08
Mn	328.61	295.44	3.08	1.37	0.34	0.13	0.11	0.03	0.14	0.03	0.26	0.14
Pb	20.35	17.11	0.28	0.1	0.23	0.1	0.08	0.04	0.09	0.04	-0.03	1.02
Se	10.17	11.24	0.03	0.01	0.89	0.58	0.27	0.12	0.55	0.49	-0.01	0.5
Zn	2989.59	2283.89	6.20	2.15	1.39	0.24	0.80	0.16	1.90	0.33	-0.65	0.77

Abbreviations: Phytoextraction (PE), Phytostabilization (PS), Translocation factor (TF), Root bioconcentration factor (BCFr), Total bioconcentration factor (BCFt), Soil decontamination (DC)

Table S3. *P*-values of all variables in the linear mixed model for each phytoremediation service. Variables with significant effect are indicated by " * " (*P*-value < 0.05) or " . " (*P*-value < 0.1).

	sp	cop	om	clay	sand	T0
**PE**	0.669	0.001 *	0.592	0.955	0.784	0.14
**PS**	0.034 *	0.000 *	0.044 *	0.274	0.606	0.684
**TF**	0.59	0.15	0.521	0.399	0.358	0.002 *
**BCFr**	0.051 .	0.849	0.021 *	0.154	0.373	0.674
**BCFt**	0.113	0.975	0.019 *	0.435	0.935	0.245
**DC**			0.324	0.073	0.207	
**DC**	**Controls**	**sp**	**1 cop**	**4 cop**		
	0.932	0.27	0.624	0.714		

Abbreviations: Phytoextraction (PE), Phytostabilization (PS), Translocation factor (TF), Root bioconcentration factor (BCFr), Total bioconcentration factor (BCFt), Soil decontamination (DC), willow diversity (sp), coppicing (cop), organic matter (OM), clay (clay) and sand (sand) content, and initial TE (T0). DC was tested for planted or not planted plots (controls), 1 or 4 willow cultivars (sp) and coppiced or not coppiced plots within the monoculture (1 cop) or polyculture (4 cop).

Table S4. The estimated slope for each variable in the models for each trait. Variables with significant effect are indicated by " * " (*P*-value < 0.05) or " . " (*P*-value < 0.1).

	**sp**	**cop**	**om**	**clay**	**sand**	**T0**
**SLA**	-0.1	1.955 *	-0.099	0.017	0.021	0.126 .
**LA**	-78.317	499.455 *	-76.841	4.700	-5.924	39.594 .
**LDMC**	11.007	-19.42 *	0.367	-0.029	0.199	-0.917
**LNC**	-0.039	0.026	0.087 *	0.005	-0.002	0.002
**LCC**	0.447	-0.069	0.235 *	0.003	-0.003	-0.076 *
**RDMC**	9.57	-10.461	1.093	-0.650	-2.282 .	-2.152
**RNC**	-0.12 *	0.13 *	0	0.000	-0.005	0.007
**RCC**	-0.878 .	-1.468 *	-0.149	0.040 .	0.057	-0.026
**SSD**	-0.012	0	-0.001	0.000	0.001	0
**LpH**	-0.056 *	-0.077 *	-0.004	0.002 *	-0.001	0.002

Abbreviations: leaf area (LA), specific leaf area (SLA), leaf pH (LpH), leaf and root dry matter content (LDMC and RDMC), leaf nitrogen and carbon content (LNC and LCC), root nitrogen and carbon content (RNC and RCC) and specific stem density (SSD), willow diversity (sp), coppicing (cop), organic matter (OM), clay (clay) and sand (sand) content, and initial TE (T0).
